# From structure to ætiology: a new window on the biology of leucine-rich repeat kinase 2 and Parkinson's disease

**DOI:** 10.1042/BCJ20210383

**Published:** 2021-07-30

**Authors:** Susanne Herbst, Patrick A. Lewis

**Affiliations:** 1 Department of Comparative Biomedical Science, Royal Veterinary College, Royal College Street, London, U.K.; 2 Department of Neurodegenerative Disease, UCL Queen Square Institute of Neurology, Queen Square, London, U.K.; 3 Aligning Science Across Parkinson's (ASAP) Collaborative Research Network, Chevy Chase, MD, U.S.A.

**Keywords:** leucine-rich repeat kinase, neurodegeneration, Parkinson's disease

## Abstract

Since the discovery of mutations in leucine-rich repeat kinase 2 (LRRK2) as an underlying genetic cause for the development of Parkinson's disease (PD) in 2004 (Neuron **44**, 601–607; Neuron **44**, 595–600), and subsequent efforts to develop LRRK2 kinase inhibitors as a therapy for Parkinson's (Expert Opin. Ther. Targets 21, 751–753), elucidating the atomic resolution structure of LRRK2 has been a major goal of research into this protein. At over 250 kDa, the large size and complicated domain organisation of LRRK2 has made this a highly challenging target for structural biologists, however, a number of recent studies using both *in vitro* and *in situ* approaches (Nature 588, 344–349; Cell 182, 1508–1518.e1516; Cell 184, 3519–3527.e3510) have provided important new insights into LRRK2 structure and the complexes formed by this protein.

Leucine-rich repeat kinase 2 (LRRK2) has proven to be a protein of interest for a number of reasons. First and foremost, the *LRRK2* locus on chromosome 12 has been either directly linked or strongly implicated in heightened risk for a range of human diseases, most notably Parkinson's disease [1–3], and most recently progressive supranuclear palsy [4]. Intriguingly, the *LRRK2* locus makes a pleiotropic contribution to the risk of Parkinson's disease with autosomal coding mutations causing Parkinson's, coding polymorphisms strongly increasing lifetime risk, and non-coding variation more subtly increasing risk as identified by genome-wide population studies [5]. This latter observation is of particular significance, as it implies that the physiological function of LRRK2 may contribute to the disease process in Parkinson's, although the functional consequences of the non-coding variants at the *LRRK2 locus* are as yet poorly understood [6]. Overall, the extant genetic data highlight LRRK2 as a potential therapeutic target not just for those with causative mutations, but also with the idiopathic form of the disorder. For fundamental biologists, the complex domain architecture and dual enzymatic activities of LRRK2 [7] (Figure 1) make it both an enthralling and an intimidating protein to study. In common with the other members of the ROCO protein family, and with the receptor-interacting protein kinases (RIPKs — with which LRRK2 is also affiliated), LRRK2 is likely to act as a signalling hub, co-ordinating and controlling myriad transduction pathways within the cell [8–10].

**Figure 1.**
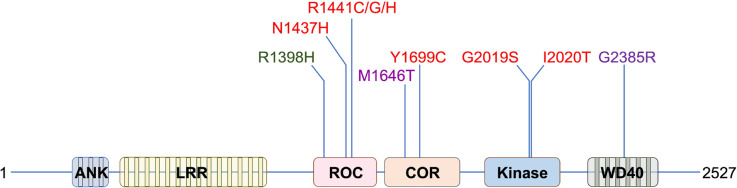
The domain organisation of LRRK2. An ideogram of LRRK2 with coding variants associated with Mendelian disease (red), increased risk of Parkinson's (purple), and decreased risk of Parkinson's (green). LRR, leucine-rich repeats; ROC, Ras of complex proteins; COR, C-terminal of ROC.

Ever since the identification of mutations in LRRK2 it has been clear that structural insights into the functions of the different domains of this protein, the interplay between them, and the impact of mutations upon these functions, would be critical for fully understanding the cellular role and pathophysiology of LRRK2. While the requirement for atomic resolution structural data relating LRRK2 was obvious, achieving this has been a herculean undertaking. This was due principally to the challenge presented by expressing and purifying such large protein (in excess of 250 kDa, and likely to be at least partially active as a dimer with the complex weighing in at over half a megadalton), a challenge perhaps exacerbated by the cytotoxic impact of expressing LRRK2 [11,12].

Initial progress on the elucidating the three-dimensional atomic resolution organisation of LRRK2 relied on either purifying isolated domains or examining orthologous proteins (or both), with crystal structures for the ROC domain of LRRK2 [13] and of the ROC–COR fragment from a prokaryotic ROCO protein from *Chlorobium tepidum* published in 2008 [14], and the kinase domain of a LRRK from the amoeba *Dictyostelium discoideum* published in 2012 [15]. A further structure for the ROC domain of LRRK2 came in 2014 [16]; however, it was not until 2016 that the first model for full-length LRRK2 was proposed, based upon a 33 Å negative stain electron microscopy reconstruction, a crystal structure for the eponymous leucine-rich repeats isolated from *C. tepidum*, and mass spectrometry mapping of interactions across the LRRK2 complex [17]. Further cryoEM analyses allowed the reconstruction of LRRK2 at 24.2 Å [18]. A structure for the WD40 protein interaction domain followed in 2019 [19], alongside a structure for the LRR–ROC–COR domains of the *C. tepidum* ROCO protein (which naturally lacks a kinase domain) [20]. These structures (summarised in Table 1 and Figure 2) provided important insights into LRRK2 function at the molecular level, but did not yield a clear picture of the mechanisms coordinating LRRK2 activities or define a consistent impact of mutations.

**Figure 2.**
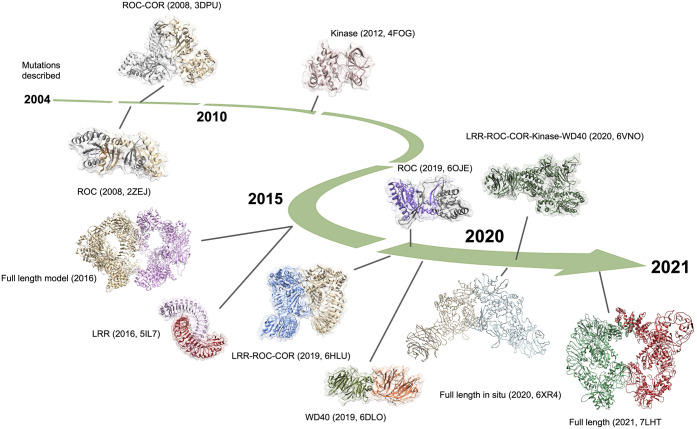
A timeline for LRRK2 structural biology. Timeline for structural advances in the LRRK2 field from the discovery of mutations in 2004 through to the present day. Details for each structure are noted in Table 1. Images generated from PDB files using Chimera [38], model for full-length LRRK2 from reference [17] courtesy of Johannes Gloeckner.

**Table 1.** Extant structures for LRRK2, including individual domains and orthologous proteins

Year	Domains	Species	Resolution	Technique	PDB	Reference
2008	ROC	*H. sapiens*	2.0 Å	X ray crystallography	2ZEJ	[13]
2008	ROC–COR	*C. tepidum*	2.90 Å	X ray crystallography	3DPU	[14]
2012	Kinase	*D. discoidium*	1.8 Å	X ray crystallography	4FOG	[15]
2016	Full length	*H. sapiens*	33 Å	CryoEM	NA	[17]
2016	LRR	*C. tepidum*	2.3 Å	X ray crystallography	5IL7	[17]
2017	Full length	*H. sapiens*	24.2 Å	CryoEM	NA	[18]
2019	ROC	*H. sapiens*	1.6 Å	X ray crystallography	6OJE	[16]
2019	WD40	*H. sapiens*	2.6 Å	X ray crystallography	6DLO	[19]
2019	LRR–ROC–COR	*C. tepidum*	3.29 Å	X ray crystallography	6HLU	[20]
2020	ROC–COR–Kinase–WD40	*H. sapiens*	3.50 Å	CryoEM	6VNO	[39]
2020	Full length	*H. sapiens*	14 Å	CryoEM	6XR4	[40]
2021	Full length	*H. sapiens*	3.7 Å	CryoEM	7LHW	[41]
2021	Full length	*H. sapiens*	3.5 Å	CryoEM	7LHT	[41]

Pathogenic LRRK2 mutations in the kinase domain (G2019S and I2020T) sit within the kinase activation loop, thereby providing a putative structural explanation for altered kinase activity. In contrast, the LRRK2 GTPase function of LRRK2 remains enigmatic, and therefore the underlying mechanisms whereby mutations in the GTPase domain influence LRRK2 activity has been harder to define.

GTPases act as molecular switches that cycle between a GDP-bound inactive state and a GTP-bound active state. In the case of small GTPases, the nucleotide cycle is controlled by guanine-nucleotide exchange factors (GEFs) which aid the exchange of GDP to GTP and GTPase-activating proteins (GAPs) which promote GTP hydrolysis [21]. However, there has been no consistent identification of GEFs or GAPs for LRRK2 and a commonly occurring Glutamine in the G3-motif of the GTPase which is required for the GTPase-GAP interaction is not conserved and instead replaced by an Arginine residue, R1398, in LRRK2. Additionally, the amino acid substitution of R1398 to Leucine, an experimental approach commonly used in small GTPase research to achieve a constitutively active GTP-bound state, results in increased GTP hydrolysis and GTPase inactivation in LRRK2 [22,23], further indicating that the mechanism of GTP hydrolysis by LRRK2 is distinct from small GTPases. Based on the homology of LRRK2 to the *C. tepidum* ROCO protein, it has been suggested that LRRK2 instead acts as a G-protein activated by nucleotide-dependent dimerisation (GAD) [24]. The GAD GTPase mechanism requires dimerisation for GTP hydrolysis whereas GEFs and GAPs are not required. Instead, residues of the protomers of a dimer pair are thought to complement each other's active site, aiding GTP hydrolysis either by stabilising the protein structure or directly providing catalytic residues [24]. Dimerisation is thought to be a requirement for LRRK2 function [25], strengthening this hypothesis. However, the composition of the dimer interface or how individual residues contribute to the catalytic mechanism remains unknown, highlighting the crucial importance of gaining structural insights into LRRK2 dimer contact sites for elucidating the LRRK2 enzymatic function.

Recently, major advances in elucidating the LRRK2 structure have been achieved. Deniston et al. [39] published a cryo-electron microscopy (EM) structure of a LRRK2 ROC–COR–Kinase–WD40 construct at a resolution of 3.5 Å, which was accompanied by an *in situ* structure of the full-length human LRRK2 I2020T mutant bound to microtubules at a resolution of 14 Å published by Watanabe et al. [40]. And more recently, Myasnikov et al. [41] resolved a full-length LRRK2 cryo-EM structure at a resolution of 3.7 Å and 3.5 Å for monomer and dimer, respectively. These structures break through the resolution limits that have hindered LRRK2 research, and expand our knowledge of the spatial relationship between the domains of LRRK2. Importantly, all three structures highlight a close proximity between the ROC–COR and the kinase domain pointing to a structural basis for the previously reported functional cross-talk between the LRRK2 ROC and kinase domains. The structures explore LRRK2 protomer interfaces in different complexes, a trimeric complex in case of the structure by Deniston et al., a multimeric helical complex in case of the *in situ* structure, and a dimeric complex for the full-length protein. All three structures identify a COR : COR domain interface mediating complex formation, with the full-length structure allowing to map individual residues of the dimer interface. The nature of the COR : COR interface is consistent with the proposed GAD mechanism for GTP hydrolysis, and was also predicted by the *C. tepidum* ROCO protein ROC–COR tandem structure. The ROCO protein structure further predicted that the GTP cycle results in conformational changes that bring the ROC domains of each dimer pair into close contact creating a ROC : ROC domain interface mediating GTP hydrolysis [14,20] (Figure 2, 3DPU). The position of the ROC domains in the dimer of the LRRK2 *in situ* and cryo-EM structures do not, however, map onto the ROCO protein models (Figure 2, 7LHTY). The ROC domains in the LRRK2 dimer structures do not come into close proximity which would be required for GTP hydrolysis aided by *trans* activation, suggesting that in the context of these structures at least, each ROC domain of LRRK2 as an independent catalytic unit. It is of note that the orientation of the ROC/COR domains is remarkedly similar to a structural model of whole human LRRK2 that integrated experimental constraints provided by chemical crosslinking [17] (Figure 2). In addition, the LRRK2 full-length structure also allows to map pathogenic mutations in the ROC–COR domain. Interestingly, the residues mutated in the ROC domain, R1441 and N1437, cluster in a helix which faces the COR domain and in particular the COR domain residue Y1699 indicating that the positioning of this helix might have an impact on the ability of LRRK2 to hydrolyse GTP.

However, comparing the structure of the LRRK2 ROC–COR–Kinase–WD40 construct to the full-length structure, the position of the ROC–COR domain in relationship to the kinase domain and WD40 differs in the full-length structure, highlighting the functional importance of the LRRK2 accessory domains. This is of particular interest in the context of the G2385R risk variant, located in the WD40 domain [26]. The arginine substitution at this residue is very clearly associated with heightened risk of developing PD in East Asian populations, but what is driving this risk at a molecular level remains unclear [27].

The recent full-length LRRK2 structure from Myasnikov and co-workers allows to map domain interfaces. Of the previously unresolved regions in LRRK2, the Leucine-rich repeat domain is particularly prominent, as it appears to shield the kinase domain from substrate entry and to stabilise a kinase-inactive conformation. Further, it acts as major hub for domain–domain interactions, by containing a helix, termed ‘hinge helix’ by Myasnikov et al*.*, that bridges the Ankyrin-repeat, Armadillo-repeat, and the WD40 domain. Additionally, the presence of the hinge helix generates steric clashes when modelled onto a previously reported WD40 : WD40 dimer structure [19]. The WD40 : WD40 interface, which was also described in the *in situ* structure, mediates the formation of large multimeric filamentous LRRK2 complexes which form around microtubules under specific experimental conditions or in specific LRRK2 mutation backgrounds. The full-length structure indicates that the position of the LRR domain is not compatible with a WD40 : WD40 homodimerization interface, at least in the reported kinase-inactive state. This raises the question as to whether the LRR domain will undergo major conformational changes upon LRRK2 activation, and under what cellular circumstances the filamentous form of LRRK2 becomes the physiologically predominant species.

Watanabe et al. focused on the LRRK2 microtubule interaction as this allowed for correlative light and electron microscopy imaging. For this form, little is known about LRRK2 kinase activity, and it is currently not clear if known LRRK2 substrates, in particular Rab GTPases, could access LRRK2 in a microtubule-bound multimeric complex. A recent study examining the role of LRRK2 in microtubule-mediated vesicle transport questions the conclusion drawn by Deniston et al., that LRRK2 might serve as a roadblock of microtubule-mediated transport. It suggests that LRRK2 facilitates anterograde transport of autophagosomes along microtubules [28]. However, Boecker et al. study LRRK2 recruited to autophagosomal membranes and its requirement for recruiting adaptor proteins of microtubule transport rather than a direct interaction of LRRK2 with microtubules. Given that recruitment of LRRK2 to endolysosomes now can be robustly achieved using chemical agents that stress lysosomal homeostasis [29,30], it is likely future LRRK2 *in situ* structures that explore this aspect of LRRK2 biology will provide significant insights. In contrast to the microtubule-bound form of LRRK2, the activation status of LRRK2 recruited to endolysosomal vesicles is better characterised, as LRRK2 membrane association is associated with kinase activity [25,31–33]. Additionally, a membrane-bound structure would be predicted to shed further light into the function and position of some of the LRRK2 accessory domains, as they are predicted to be involved in membrane association.

An endomembrane-bound structure might also help to overcome the next hurdle for LRRK2 biology: capturing the protein in a kinase active conformation at a higher resolution than the previous *in situ* structure allowed for. LRRK2 being captured in an inactive conformation in the full-length cryo-EM structure could be a consequence of the structure being solved in a GDP-bound inactive state. GTP-binding is thought to mediate intra- and inter-domain movements as seen for other ROCO proteins and directly affects LRRK2 kinase activity [20]. Additionally, recent work indicated that a ROCO ROC–COR construct dimerises in a GDP-bound state and that GTP-binding results in monomerization and GTP hydrolysis [34]; however, it is unclear to date if a similar GDP/GTP cycle would apply to the full-length LRRK2 protein. It is tempting to speculate that elaborating on the current structure in a GTP-bound state would provide pivotal clues to further illuminate LRRK2 function and the roles that specific residues might play therein. Notably, the active/inactive conformation of LRRK2 will be of relevance to our understanding of how type I and type II kinase inhibitors interact with and modulate LRRK2 [35]. Elucidating the dynamic interplay between GDP-bound and GTP-bound states, and monomeric and dimeric protein conformations remains a challenge in structural biology that is acutely relevant in the case of LRRK2.

Taken together, these LRRK2 structures present a major step forward for the field, facilitating the generation of testable structure-based hypotheses for LRRK2 biology and pathobiology. At the same time, they act to highlight the limits of our understanding of the molecular function of this protein. The most recent full-length structure has revealed complex LRRK2 inter-domain interactions, further clarified the mechanistic impact of the LRRK2 kinase mutations but stopped short in eluding the underlying mechanism of the LRRK2 ROC–COR domain mutations. Therefore, gaining further insights into the connection between structure and function will be critical to refine and develop ongoing efforts to target LRRK2 in Parkinson's, for example by illuminating the allosteric consequences of kinase inhibition and the distinctions between structurally dissimilar compounds [36]. In addition, testing whether there is a convergent structural and biochemical impact of the different coding mutations and risk variants in LRRK2 linked to Parkinson's disease is still, 17 years after the first description of mutations in LRRK2, a key priority for the field [37]. As new structures become available building on these recent successes, they will provide hope that we are entering a new phase of LRRK2 research where this may, at last, be possible.

## Open Access Statement

Open access for this article was enabled by the participation of University College London in an all-inclusive *Read & Publish* pilot with Portland Press and the Biochemical Society under a transformative agreement with JISC.

## Abbreviations

EM: electron microscopy
GAD: G-protein activated by nucleotide-dependent dimerisation
GAPs: GTPase-activating proteins
GEFs: guanine-nucleotide exchange factors
LRRK2: leucine-rich repeat kinase 2
PD: Parkinson's disease
